# The Function of H2A Histone Variants and Their Roles in Diseases

**DOI:** 10.3390/biom14080993

**Published:** 2024-08-12

**Authors:** Xuemin Yin, Dong Zeng, Yingjun Liao, Chengyuan Tang, Ying Li

**Affiliations:** 1 Department of Nephrology, The Second Xiangya Hospital, Central South University, Changsha 410011, China; 228211029@csu.edu.cn (X.Y.); 238202059@csu.edu.cn (D.Z.); lyjundream@csu.edu.cn (Y.L.); tangchengyuan@csu.edu.cn (C.T.); 2 Hunan Key Laboratory of Kidney Disease and Blood Purification in Hunan Province, Changsha 410011, China

**Keywords:** histone variants, chromatin, H2A.Z, H2A.B, macroH2A, H2A.X, gene transcription, DNA damage repair, cancer, embryonic development abnormalities

## Abstract

Epigenetic regulation, which is characterized by reversible and heritable genetic alterations without changing DNA sequences, has recently been increasingly studied in diseases. Histone variant regulation is an essential component of epigenetic regulation. The substitution of canonical histones by histone variants profoundly alters the local chromatin structure and modulates DNA accessibility to regulatory factors, thereby exerting a pivotal influence on gene regulation and DNA damage repair. Histone H2A variants, mainly including H2A.Z, H2A.B, macroH2A, and H2A.X, are the most abundant identified variants among all histone variants with the greatest sequence diversity. Harboring varied chromatin occupancy and structures, histone H2A variants perform distinct functions in gene transcription and DNA damage repair. They are implicated in multiple pathophysiological mechanisms and the emergence of different illnesses. Cancer, embryonic development abnormalities, neurological diseases, metabolic diseases, and heart diseases have all been linked to histone H2A variant alterations. This review focuses on the functions of H2A histone variants in mammals, including H2A.Z, H2A.B, macroH2A, and H2A.X, and their current roles in various diseases.

## 1. Introduction

Chromatin is essential in the transmission of genetic information and vital biological processes, such as DNA replication, DNA damage repair, and gene transcription. The fundamental functional constituent unit of chromatin is the nucleosome, which comprises DNA and a histone octamer. In multicellular organisms, histone H1 unites two nearby nucleosomes, preserving higher-order chromatin structure [1]. Generally, the histone octamer consists of two H2A-H2B dimers and one H3-H4 tetramer, also known as the canonical histone. Unlike canonical histones that are encoded by multiple genes, histone variants, also known as noncanonical histones, are usually encoded by single gene [2]. Structurally, the canonical histone mRNAs lack introns and polyadenylation as well as contain stem-loop structures at their 3’ends in mammals, while the histone variant mRNAs are poly-adenylated [3]. Throughout the S-phase of the cell cycle, canonical histones are abundantly expressed and assembled onto chromatin upon DNA replication. In contrast, histone variants are assembled onto chromatin throughout all stages of the cell cycle [4]. This feature of histone variants creates conditions for flexible regulation of gene transcription. In eukaryotes, DNA is packed tightly with nucleosomes, maintaining a small space in the nucleus. However, gene transcription and DNA replication necessitate the loose binding of the DNA and histone octamers [5]. Due to the different structures and features compared to canonical histones, histone variants can change the binding state between DNA and histone octamers, and modify chromatin structure by replacing canonical histones. Histone variants replace canonical histones at several specific regions on chromatin, such as the gene promoter and enhancer regions [6,7,8,9]. These regions are important for the regulation of gene transcription, thus showing the importance of histone variants for gene regulation. In recent years, histone variant regulation has been valued as an important form of epigenetic regulation [10,11]. Considering the important effect of histone variants on gene transcription, it is essential to study histone variant regulation.

When assembling into chromatin, histone variants require the assistance of histone chaperones. These chaperones reduce histone aggregation and prevent the non-specific binding of positively charged histones with negatively charged DNA [12,13]. Histone chaperones usually are involved in disease onset and progression by influencing histone variants [14]. In addition to being involved in gene transcription, histone variants have considerable effects on DNA damage repair [15,16]. Histone variants include histone H2A variants, histone H2B variants, histone H3 variants, and histone H4 variants. Histone H2A variants are the most abundantly identified variants with the greatest sequence diversity [17]. Therefore, we focus on the functions and roles of histone H2A variants. Histone H2A variants typically differ from H2A histones in their C-terminus, which contributes to nucleosome stabilization, substitution, and DNA binding to regulators, laying the structural foundation for their functional differences [18,19,20]. The structures of histone H2A variants and the relationships between their structures and functions are described in a separate review [8]. The onset and progression of diseases are intricately linked to gene regulation. Histone variant regulation, as a crucial mechanism for controlling gene transcription, has garnered increasing attention in the study of various diseases in recent years, such as cancer, embryonic development abnormalities, neurological diseases, metabolic diseases, and heart diseases. Understanding their roles in diseases could provide valuable insights into the effective treatment of diseases. This review provides a comprehensively analysis of the functional roles of histone H2A variants, including H2A.Z, H2A.B, macroH2A, and H2A.X, and their associations with various diseases in mammals.

## 2. The Functions of H2A Histone Variants

### 2.1. Gene Transcription

**Figure 1:**
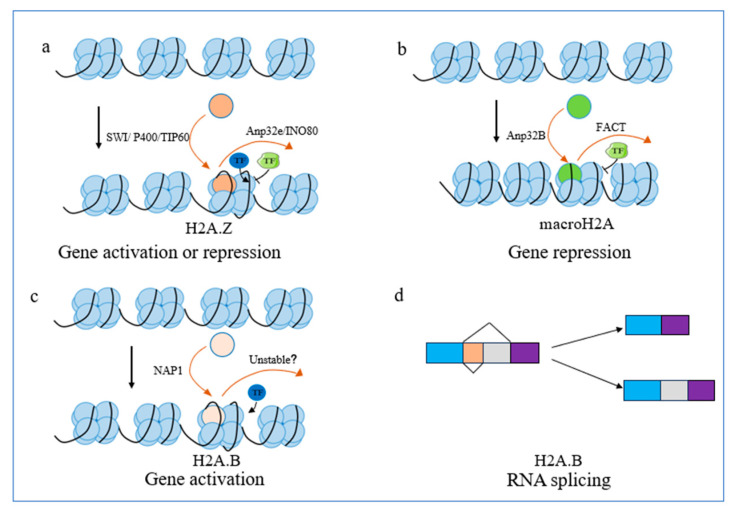
The role of H2A histone variants in gene expression. (**a**) With the help of SWI/P400/TIP60, histone variant H2A.Z is incorporated into chromatin increasing the bind of transcription factors with opening chromatin, thus repressing or promoting gene transcription. Histone chaperones Anp32e and INO80 participate in the removal of H2A.Z in chromatin. TF refers to transcription factors. (**b**) The main function of macroH2A is to repress gene expression, which is associated with chromatin condensation. Histone chaperone Anp32B and FACT participate in the incorporation and removal of macroH2A in chromatin. (**c**) With the assistance of histone chaperone NAP1, histone variant H2A.B is incorporated into chromatin, loosening the compact chromatin structure and increasing the bind of transcription factors with DNA. However, whether the histone chaperones mediate the removal of H2A.B from chromatin is still unclear. (**d**) H2A.B facilitates gene expression by regulating RNA splicing.

H2A.Z plays a dual role in gene transcription, acting as an activator and a repressor (Figure 1a). H2A.Z contains two isoforms in vertebrates, known as H2A.Z1 and H2A.Z2 [21]. With the advancement of biotechnology, the third isoform H2A.Z2.2 has been discovered in humans [21,22,23]. Although H2A.Z2 differs from H2A.Z1 by only three amino acids, this difference dramatically affects its function. Compared with H2A.Z1, H2A.Z2 deposits on AT-rich enhancers and is more susceptible to SNF2-related CBP activator protein (SRCAP) deletion. Increasing H2A.Z2 alleviates symptoms in patients with Floating-Harbor syndrome with the SRCAP gene mutation [24]. The spatial distribution of H2A.Z within the chromatin landscape establishes the necessary conditions for its function. Studies show that H2A.Z is widely distributed throughout the genome, especially promoter and enhancer regions, and is essential to nucleosome turnover, DNA repair, heterochromatin formation, and gene transcription [21,25,26]. Histone chaperones regulate the location of H2A.Z in chromatin. Acidic nuclear phosphoprotein 32 family member E (Anp32e) and chromatin remodeling complex inositol-requiring mutant 80 (INO80) mediate the dissociation of H2A.Z-H2B from nucleosomes in the promoter region and especially the enhancer and insulator regions, whereas SWR1 in *yeast* and p400/TIP60 in mammals regulate H2A.Z deposition in chromatin [27,28]. The mechanism through which H2A.Z modulates gene transcription is complex, and to date, we have yet to fully comprehend the precise manner in which H2A.Z influences gene transcription. Rudnizky et al. observed that the substitution of nucleosomes at the +1 position with H2A.Z resulted in a diminished stability of nucleosomes and an enhancement of transcriptional efficiency, promoting gene transcription [29]. Consistent with this result, Li et al. discovered that incorporating H2A.Z into chromatin reduces the energy required for local chromatin wrapping and increases DNA accessibility [30]. Another study showed that the deposition of H2A.Z in the genome was positively correlated with the suspension of RNA polymerase II by CHIP-seq [31]. The accumulated evidence suggests that H2A.Z is involved in the activation of gene regulation. However, there remains a gap in our understanding regarding its role in repressing gene transcription. Current research has implied that the repression function is closely related to the location of H2A.Z on chromatin and whether it exists in a stable or unstable form [32]. H2A.Z removal upstream of the Transcription Start Site (TSS) inhibits epithelial gene expression, whereas its removal downstream of the TSS stimulates mesenchymal gene expression [33]. The findings of this study illustrate that the repression of H2A.Z at distinct TSS locations resulted in different effects on gene transcription. Nonetheless, this observation does not elucidate the underlying mechanisms responsible for these distinct effects. Intriguingly, a recent scholarly article explains the diverse functions of H2A.Z based on its structural attributes. Lewis et al. constructed nucleosomes using canonical *Xenopus* H2A and mouse H2A.Z1 in vitro to mimic the incorporation of the histone H2A.Z variant into chromatin in vivo. Cryo-EM structure reveals that H2A.Z improves DNA end mobility, reduces the stability of nucleosomes, and hence stimulates gene transcription. This function is primarily attributed to the short C-terminal domain of H2A.Z [34]. A previous crystallography study showed DNA encapsulates the H2A.Z octamer in a similar manner to the canonical histone octamer, but with subtle differences in the nucleosome surface around the acidic patches [35]. The acidic patches present on the H2A.Z histone variant nucleosomes allow nucleosomes to form denser chromatin fibers. In the context of heterochromatin, the consecutive accumulation of multiple H2A.Z nucleosomes leads to the compaction of chromatin structure, which in turn promotes gene repression [34]. Furthermore, we speculate the dual functionality of H2A.Z might be elucidated by the stability of nucleosomes in diverse biological contexts. H2A.Z nucleosome stability varies depending on different chromatin contexts, such as histone variants Post-Translational Modifications (PTMs), chromatin regulators, and the presence of other histone variants [25,32]. This provides a basis for different roles of H2A.Z in different contexts. Interestingly, several histone chaperones regulate gene expression by targeting H2A.Z. Anp32e modulates the phosphorylation of H2A.Z through protein phosphatase 2A (PP2A), thus altering gene transcription to induce growth in the heart [36]. The methyltransferase SET and MYND domain-containing protein 3 (SMYD3) enables H2A.Z1 methylation, thereby reducing the removal of H2A.Z1 from chromatin by Anp32e, increasing cell cycle-related protein expression, and promoting breast cancer cell proliferation [37]. In *yeast*, histone deacetylase Rpd3 large (Rpd3L) complexes play a crucial role in regulating INO80 protein levels via triggering deacetylation. This hinders the expression of autophagy-associated genes by dissociating H2A.Z from autophagy-associated chromatin [38]. Those results suggest the importance of histone chaperones in targeting histones and regulating their functions in biological processes cannot be overstated. However, whether different histone chaperones work in synergy remains a subject for further investigation. In conclusion, the role of H2A.Z in gene regulation is complex, and the mechanism underlying why distinct H2A.Z loci generate opposite gene transcription effects is unknown. Moreover, how the factor network mediates gene transcription by targeting H2A.Z remains to be further explored.

H2A.B facilitates gene transcription activation (Figure 1c,d). It demonstrates the highest expression in the mammalian testes, with subsequent notable expression observed in the brain [39]. Histone chaperone Nucleosome Assembly protein 1 (NAP1) interacts with H2A.B and promotes its deposition in chromatin [40]. However, no studies have shown that histone chaperones mediate H2A.B dissociation from nucleosomes. Particularly, studies have shown that H2A.B nucleosomes are poorly stable, allowing them to easily dissociate from chromatin, forming open chromatin structures and regulating gene expression [19,20]. Nevertheless, whether H2A.B has histone chaperones mediating its dissociation from chromatin deserves further exploration. Three non-allelic genes, namely *H2AB1*, *H2AB2*, and *H2AB3*, have been identified in encoding the histone variant H2A.B [19,41]. The location of H2A.B on chromatin is associated with its regulation of gene activation. In mice, the homologous analog of H2A.B is H2A.lap1 (H2A.B3), which is situated in the TSS region and is positively related to gene transcription activation [42]. Another study in the mouse hippocampus confirms this conclusion [39]. Nucleosome instability is the primary mechanism of H2A.B regulating gene transcription [19,20]. Cryo-EM structure reveals that H2A.B nucleosomes are poorly stable, permitting relatively easy dissociation from chromatin, the formation of open chromatin structures, and gene transcription activation. This is primarily due to the absence of the C-terminus of H2A.B [19,20]. However, nucleosome stability does not fully explain the gene regulation of H2A.B. Interestingly, molecular dynamics simulation shows that H2A.B diminishes protein–protein and protein–DNA interactions, and leading to enhanced DNA accessibility in chromatin [43]. This further explains the mechanism by which H2A.B affects gene transcription. In addition, H2A.B has been reported to modify the RNA alternative splicing process [39,44], which is an essential process in post-transcriptional regulation [45]. H2A.B recruits splicing regulators and RNA polymerase II in active genes [39]. Consistent with this result, male mice with knockout *H2A.B3* show a reduction in RNA polymerase II and altered splicing factors [44]. Therefore, we speculate that H2A.B is likely to promote gene expression by regulating the variable shearing of RNA. In conclusion, H2A.B has been identified as a regulator that facilitates gene transcription. However, the current research on H2A.B is limited. It is crucial to elucidate the underlying mechanisms by which H2A.B modulates gene transcription. Could there be regulation of H2A.B by PTMs? What gene regulatory role does H2A.B play in different tissues? Apart from activating gene transcription, does H2A.B have other effects? Interestingly, studies show that H2A.B is widely present in DNA damage sites and replication sites [46,47]. The overexpression of H2A.B in Hela cells has been shown to induce DNA damage and subsequent apoptosis by activating the Nuclear Factor-Kappa B(NF-κβ) pathway [48]. The evidence suggests that H2A.B potentially plays a role in repairing DNA damage. The exact way H2A.B contributes to this process warrants deeper exploration.

MacroH2A has been proven to inhibit gene expression (Figure 1b). MacroH2A is a widely distributed and evolutionarily conserved protein in a diverse variety of vertebrate species [49]. A genome-wide investigation revealed that macroH2A is genetically widespread and highly abundant in transcriptionally silent regions of chromatin associated with Histone 3 Lysine 27 trimethylation (H3K27me3). Active genes typically lack macroH2A, and macroH2A deficiency reduces dense chromatin [50]. The position of macroH2A on chromatin plays a critical role in modulating its ability to suppress gene transcription. Facilitates Chromatin Transcription (FACT) is responsible for removing macroH2A from transcriptionally active regions [3]. Aprataxin and polynucleotide kinase-like factor (APLF) bind to macroH2A and deposit it into chromatin in reaction to DNA damage [51]. A recent study identified a novel macroH2A histone chaperone in humans: Acidic Nuclear Phosphoprotein 32 Family Member B (Anp32B). Anp32B interacts with macroH2A and regulates macroH2A deposition in chromatin [52]. Histone chaperone regulation allows macroH2A to flexibly alter chromatin structure. The non-allelic genes *MACROH2A1* and *MACROH2A2* encode for macroH2A1 and macroH2A2, respectively. *MACROH2A1* produces two different transcripts, so there are two other proteins, macroH2A1.2 and macroH2A1.1 [9]. The mechanism by which macroH2A inhibits gene transcription is complicated and has not yet been fully elucidated. In nucleosomes incorporating macroH2A, nucleosome remodeling activity is inhibited, and the mobility of the histone octamer is constrained [53,54]. In addition, macroH2A repressing gene transcription is related to chromatin condensation in constitutive heterochromatin that maintains genomic stability and ensures stable gene replication by blocking the transcription of transposons and simple DNA repeats [50]. The result has been further proven by the fact that the deletion of macroH2A leading to structural alterations in constitutive heterochromatin, thus reducing gene stability [55]. Interestingly, macroH2A can affect the level of chromatin acetylation and thus repress gene expression [53,56]. This approach is independent of its direct effect on chromatin structure. It is worth noting that while the majority of research on macroH2A has centered around its role in gene repression, there is also evidence to support its association with gene activation. MacroH2A contributes to maintaining the balance of activating/repressing chromatin remodeling complexes, thereby regulating gene activation and repression [57]. In addition, macroH2A possesses the ability to mask repressor binding sites in TSS region of expressing genes to perform gene activation functions [58]. Notably, a recent study has suggested that macroH2A1 induces the release of paused RNA polymerase II to enhance gene expression in triple-negative breast cancer cells [59]. This ambivalent function of macroH2A may explain both its promotion and inhibition roles in cancer proliferation, as described below. These results above partially explain the mechanism by which macroH2A exerts its dual action. However, how macroH2A structure affects its dual function, just like H2A.Z, remains unclear. Furthermore, most current research on macroH2A has been focused on its role in suppressing gene transcription. However, further investigation is required to understand its mechanisms in gene activation.

### 2.2. DNA Damage Repair

**Figure 2:**
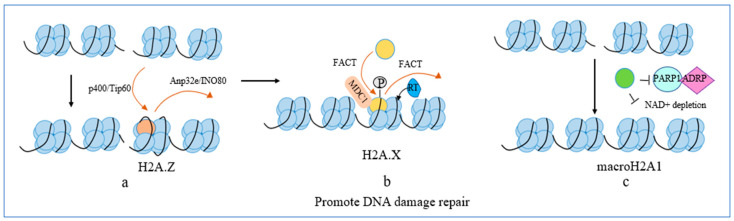
The role of histone H2A variants in DNA damage repair. (**a**) H2A.Z promotes DNA damage repair by eviting nucleosomes in chromatin with the help of the histone chaperones Anp32e and INO80. (**b**) γ-H2A.X accelerates DNA damage repair by recruiting DNA damage repair factors. RT refers to repair factors. (**c**) MacroH2A1 impedes the activity of PARP1 and prevents depletion of NAD+, thereby promoting DNA damage repair.

H2A.Z is a prominent modulator of gene transcription (Figure 2a), and its role in DNA damage repair has also been demonstrated [60,61,62]. H2A.Z is also enriched in the DNA damage region [62]. In response to DNA damage, histone variant H2A.Z undergoes dynamic changes. During the initial period of DNA damage repair, H2A.Z is deposited into the chromatin with the aid of p400/Tip60 [60]. Subsequently, H2A.Z is dissociated from chromatin with the assistance of histone chaperones Anp32e and INO80, which alters the acidic patches on the nucleosome surface and promotes acetylation of the H4 tail, leading to a change in chromatin from an inhibited state to an open state, thereby recruiting repair factors to the break region and promoting DNA damage repair. Co-depletion of H2A.Z reverses DNA damage repair failure after the knockdown of Anp32e or the INO80 chromatin remodeling complex (INO80) [61,63]. These findings suggest that the exchange of H2A/H2A.Z at regions of DNA damage is the primary mechanism by which H2A.Z contributes to DNA damage repair. A recent study has further demonstrated that the FBXL10-RNF68-RNF2 ubiquitin ligase complex (FRRUC) mediates H2A monoubiquitylation, facilitating the exchange of H2A/H2A.Z at regions of DNA damage and mediating DNA damage repair [64]. These discoveries further emphasize the significant involvement of histone variant regulation in DNA damage repair. Consequently, it is implied that H2A.Z participates in DNA damage repair through the mechanisms that are independent of gene transcription regulation. However, recent studies have shown that gene transcription is closely related to DNA damage repair [65,66], which raises the question of whether H2A.Z can contribute to DNA damage repair by influencing gene transcription. In conclusion, although H2A.Z is not the most critical molecule for DNA damage repair, it serves as the fundamental basis for repairing DNA damage (Figure 2a). Ubiquitination modifications are involved in the dissociation of H2A.Z from chromatin. It is worth investigating whether other PTMs play a role in the exchange of H2A/H2A.Z in response to DNA damage repair. In addition, whether H2A.Z mediates DNA damage repair by affecting gene transcription deserves further exploration.

H2A.X is a DNA damage repair factor (Figure 2b). The mechanism of H2A.X in DNA damage repair has been extensively researched. The H2A.X mutation compromises DNA repair efficiency, enhancing sensitivity to radiation and genotoxic agents [67]. A genome-wide analysis reveals that H2A.X is constitutively integrated across the genome at baseline, and it is mainly localized in the TSS region, where replication is susceptible to DNA damage [68]. It has been shown that Anp32e first mediates the dissociation of H2A.Z in chromatin [69]. Then, the FACT complex facilitates the deposition of newly synthesized H2A.X onto nucleosomes at sites of DNA damage, leading to the displacement of H2A nucleosomes by H2A.X, which further boosts the recruitment of DNA damage repair factors and promotes DNA damage repair [69,70]. DNA damage detection induces local H2A.X S139 phosphorylation (γ-H2A.X) activated by PI3K-like-kinases [67]. Then, γ-H2A.X interacts with MDC1, further stimulating PI3K-like-kinase activity and consequently increasing the phosphorylation of H2A.X [6,71]. This suggests that γ-H2A.X amplifies the signal and functions as a platform for assembling the DNA damage repair machinery. In addition to the classical phosphorylation of the H2A.X S139 site, H2A.X Tyr142 is discovered to participate in DNA damage repair. WSTF, a component of the WICH ATP-dependent chromatin remodeling complex, mediates the phosphorylation of H2A.X Tyr142, speeding up DNA damage repair in mammals [72]. This suggests that H2A.X phosphorylation at different sites can contribute to DNA damage repair. Additionally, ubiquitination is also involved in DNA damage repair [73,74,75]. Ubiquitinating enzymes RNF8 and RNF168 target H2A/H2A.X to mediate DNA damage repair signaling [76]. Under normal cellular conditions, Histone Deacetylase 6 (HDAC6) binds to H2A/H2A.X, thereby inhibiting RNF168 from binding to H2A/H2A.X. Following DNA damage, RNF168 rapidly ubiquitinates the lysine 116 site of HDAC6, leading to the degradation of HDAC6. This degradation allows RNF168 to bind to and ubiquitinate H2A/H2A.X, consequently prompting the recruitment of DNA double-strand break (DSB) repair factors (53BP1 and BRCA1) to the chromatin [74]. The above results suggest that PTMs of H2A.X play a vital role in DNA damage repair. Interestingly, H2A.X has been shown to form a stable complex with Poly ADP-Ribose Polymerase 1 (PARP1), a crucial detector for DNA damage, boosting PARylation and facilitating DNA damage repair under diminished NAD+ levels [77]. This finding provides new insights into the mechanism by which non-PTM-modified H2A.X is involved in DNA damage repair. In addition, it has been shown that H2A.X forms a complex with Apoptosis-Inducing Factor (AIF) and cyclophilin A in the nucleus under apoptosis states [78,79,80]. AIF acts as a nuclease enzyme and promotes the degradation of double-stranded DNA [78]. Reducing the expression of H2A.X attenuated the mediated chromatinolysis effect of AIF [79]. This suggests that H2A.X plays an important role in chromatinolysis. H2A.X is a primary DNA damage repair regulator, while a recent study shows that the phosphorylation H2A.X axis mediates TGFB1-associated gene transcription activation, thereby aggravating pulmonary fibrosis [81]. In addition, γ-H2A.X regulates the self-renewal and differentiation of human pluripotent stem cells and leukemic progenitors [82]. These observations underscore the critical function of H2A.X in gene regulation. Yet, the significance of H2A.X in gene transcription remains underexplored, calling for deeper investigation in future research.

MacroH2A promotes DNA damage repair (Figure 2c). Mechanistically, two distinct isoforms of macroH2A, namely macroH2A1.2 and macroH2A1.1, facilitate chromatin compaction and repress its expansion after DNA damage. Transcriptional repression is important in early DNA damage repair. MacroH2A1.2 and macroH2A1.1 are required for transcriptional repression near the break areas following DNA damage [50,83,84], and their unstructured linker region is crucial for repressive function [83]. Additionally, they are involved in DNA damage repair in different ways. MacroH2A1.2 targets DNA damage repair in a homologous recombination pathway by recruiting DNA damage repair mediator BRCA in the DNA damage region [84], while macroH2A1.1 mediates DNA damage repair mainly through targeting PARP1 [83,85]. It has been demonstrated that the macro domain of macroH2A1.1 engages in competitive binding with PARP1 for ADP-ribose (ADPR), impeding the activity of PARP1 and diminishing the transient PARP1-mediated chromatin relaxation in the context of DNA damage [83,85]. In addition, macroH2A1.1 prevents the depletion of NAD+, a substrate for ADRP, and lowers cell mortality caused by PARP overactivation, hence promoting DNA damage repair [86]. Furthermore, it is noteworthy that different isoforms of macroH2A1 perform diverse functions in gene regulation, possibly related to their different affinity bindings to PARP1. MacroH2A1.1 regulates gene transcription by targeting PARP1 [87,88], whereas macroH2A1.2 regulates transcription by recruiting transcriptional regulators [89]. Most scientific studies focus on exploring the competitive binding of macroH2A1.1 to PARPs and its significance in DNA damage repair. However, a recent article has brought to light the existence of a functional association between macroH2A1.2 and PARPs [90]. PARP1 promotes Poly (ADP-Ribose) (PAR) synthesis and recruitment of lysine-specific demethylase 5A (KDM5A) to DNA damage sites upon the occurrence of DNA damage. Furthermore, macroH2A1.2 also facilitates the accumulation of KDM5A at DNA damage repair. The concerted effort of PARP1 and MacroH2A1.2 promotes the repair of DNA damage by facilitating the recruitment of KDM5A [90]. Interestingly, the involvement of macroH2A in DNA damage repair is centered on two isoforms of macroH2A1. However, Y et al. found that the lack of 15-LOX-1 in colorectal cancer downregulates macroH2A2 expression and decreased macroH2A2 expression inhibits DNA damage repair by delaying H2A.X activation [91]. The macro-domain region of macroH2A serves the function of binding ADP-ribose [92], and this could potentially explain why they both fulfill crucial roles in DNA damage repair. This article exposes the role of macroH2A2 in DNA damage repair for the first time. The prominent role of macroH2A2 is to repress gene transcription. Whether macroH2A2 is also involved in chromatin compaction during the initial stages of DNA damage repair deserves further exploration.

## 3. The Roles of Histone H2A Variants in Diseases

Histone variants are known to play a crucial role in influencing the onset and progression of numerous diseases by modulating gene transcription and DNA repair. It has been proven that histone H2A variants are essential in cancer [93,94], embryonic development-related diseases [95,96,97], neurological diseases [97,98], muscle regeneration disorders [99], cellular senescence [100,101], metabolic disorders [102,103,104], and cardiovascular diseases [105,106,107]. Among these diseases, cancer and embryonic development-related diseases are the most extensively studied. Distinct histone H2A variants have been found to fulfill different functions in diverse diseases.

### 3.1. Cancer

Histone H2A variants are implicated in cancer (Table 1 and Table 2). Cancer continues to be a major global health concern with a significant impact on populations worldwide. According to the International Agency for Research on Cancer (IARC), in 2022, there were almost 20 million new cases of cancer and approximately 9.7 million cancer-related deaths globally [108]. Cancer is a complex illness defined by genetic instability generated by both oncogenic mutations and a variety of external and endogenous causes. This instability leads to alterations in the cellular genome, resulting in uncontrolled cell proliferation [109]. Histone H2A variants play a crucial role in tumor development by governing gene transcription and DNA damage repair. PTMs of histone H2A variants have been implicated in cancer pathogenesis [93,110,111]. Different histone variants exhibit distinct roles in cancer development and progression. H2A.Z is commonly recognized as a pro-oncogene in a variety of cancer types. As a marker of DNA damage repair, H2A.X is considered to harbor anticancer properties. MacroH2A has a key function in carcinogenesis, acting as both an oncogene and a tumor suppressor. Nevertheless, there is limited research on the function of H2A.B in cancer. According to one study, H2A.B is enriched in the rDNA promoter region, interacts with RNA polymerase II, boosts ribosome production in tumor cells, and promotes tumor development in Hodgkin’s lymphoma [112]. Interestingly, a recent paper has shown that H2A.B expression is abnormally upregulated in a range of cancers, including endometrial and urothelial bladder carcinomas, implying a potential role for H2A.B in cancer [113]. Similarly, H2A.Z, H2A.X, and macroH2A are associated with the modulation of the Epithelial-Mesenchymal Transition (EMT) process [114]. This crucial event confers cancer cells with stem cell properties and promotes tumorigenesis, thereby exerting a significant influence on tumorigenesis [115,116]. The detailed roles of H2A.Z, H2A.X, and macroH2A in cancer are described below.

H2A.Z is identified as a pro-oncogene in various cancer types, including lung [117], breast [37], uterine [118], liver [110], malignant melanoma [119], and bladder cancers [120]. The roles of H2A.Z1 and H2A.Z2 in cancer are distinct, though only with several amino acids being different. Tang et al. reported that hepatocellular cancer patients exhibited significantly elevated levels of H2A.Z1 and H2A.Z2. Both proteins were correlated with a poor prognosis. RNA-seq revealed that H2A.Z1 regulates RNA splicing, while H2A.Z2 is implicated in the spindle midzone and microtubule [121]. It was shown that removing H2A.Z1 triggers EMT, but H2A.Z2 has no effect on EMT [33]. This conclusion is also obtained in lung cancer, where H2A.Z.1 has been found to enhance the sensitivity of lung cancer cells to radiotherapy by targeting EMT [117]. The distinct function may be due to their differences in SRCAP sensitivity [24]. Furthermore, H2AZ2 and H2A.Z1 are studied in different cancer types. H2A.Z2 is most thoroughly investigated in malignant melanoma. The lack of H2A.Z2 in malignant melanoma enhances tumor sensitivity to drugs. The underlying mechanism is related to its gene transcriptional regulation for E2F1 [119]. H2A.Z1 enhanced tumorigenesis in hepatocellular carcinoma cells, mostly through cell cycle signaling and the DNA damage pathway. It has been shown that Tumor Protein 53 (TP53) induces H2A.Z1 overexpression, which facilitates hepatoma cell proliferation [122]. In addition, PTMs play an essential role in the regulation of tumors. CHIP-seq shows a high acetylation of H2A.Z in liver cancer cells. LincRNA ZNF337-ASI enhances the acetylation of H2A.Z, boosting downstream pro-oncogene transcription in liver cancer [110]. SMYD3 suppresses the methylation of H2A.Z1, thereby inhibiting Anp32e-mediated dissociation of H2A.Z1 from chromatin, increasing cyclin A1 expression, and encouraging breast cancer progression [37]. While the majority of studies have posited H2A.Z as a pro-carcinogenic factor, a recent study has revealed the deficient H2A.Z disposition in uterine leiomyoma cells with SRCAP complex mutations, which suggests a cancer inhibition role of H2A.Z in tumorigenesis [118]. The study’s findings provide valuable insights into the mechanism involvement of H2A.Z variants in uterine leiomyoma and offer new avenues for further research. It is noteworthy that different H2A.Z variants play distinct roles in tumors, which raises the question of whether there are effective ways to regulate the expression of different H2A.Z variants to inhibit tumor development. In brief, H2A.Z is a crucial factor in the development of tumors and is considered an effective target for therapy. While extensive research has been conducted on H2A.Z in tumors, many questions remain unanswered, such as the role of H2A.Z in tumors when mutations are present and how to regulate H2A.Z isoforms.

H2A.X is considered to harbor anticancer properties in gastrointestinal cancer [123,124], breast cancer [125,126], prostate cancer [111], head and neck carcinoma [127], and leukemia [128]. DNA damage repair plays a decisive role in cell proliferation, cancer development, and cancer treatment [129]. H2A.X mediates cancer development as a key molecule regulating DNA damage repair. It has been shown that ROCK-1 causes DNA breaks and cell cycle arrest via inhibiting the H2A.X/H2B-p21 axis, leading to irreversible DNA damage and apoptosis in lung cancer cells, thus favoring a promising target for the treatment of lung cancer [130]. H2A.X is a widely recognized biomarker of DNA damage repair in various types of cancer. In recent research, such as the study of adavosertib in conjunction with ricolinostat treatment for head and neck cancer, the detection of H2A.X expression has been utilized to effectively assess the extent of DNA damage repair in cancer cells [131]. In addition to acting as a DNA damage marker to regulate tumorigenesis and progression, could H2A.X be involved in tumorigenesis by other means? Genome-wide expression analysis showed that deletion of H2A.X leads to an increase in the expression of EMT-related genes in breast cells. Further experimental validation confirmed that enhanced Twist1 and Slug transcription factors regulate EMT and facilitate tumor cell migration and invasion after H2A.X deletion in breast cancer [125]. In line with breast cancer, lowering H2A.X triggers EMT in colorectal adenocarcinoma cells [123]. These results suggest that H2A.X can mediate the expression of EMT genes involved in the regulation of tumorigenesis, which provides new evidence for the involvement of H2A.X in gene regulation. Furthermore, PTMs of H2A.X have been identified as a crucial component in the pathophysiology of tumorigenesis. Li et al. reported that knocking down double-stranded RNA-specific adenosine deaminase (ADAR1) improves cancer cell death, increases H2A.X phosphorylation, and suppresses prostate cancer cell growth [111]. AKTs promote the Ser19 site phosphorylation of H2A.X and thus suppress the survival and metastasis of breast cancer cells [126]. Phosphorylation of H2A.X Y142 has a vital role in DNA damage repair, and a study has shown that livin can increase autophagy and inhibit cancer development in colorectal cancer cells by promoting the phosphorylation of H2A.X Y142 [124]. It is worth noting that γ-H2A.X can be utilized as a non-invasive technique for predicting carcinogenesis because it is easily identified in human peripheral blood, and cancer patients are typically diagnosed in the absence of H2A.X [132,133]. As a result, detecting γ-H2A.X is regarded as a valuable method for cancer prediction. In conclusion, H2A.X is an important oncogenic molecule. Current studies have focused on H2A.X’s role in mediating cancer development by affecting DNA damage repair, and more research is needed in the future to investigate its mediation of tumorigenesis through non-DNA damage repair mechanisms.

MacroH2A is critical to tumorigenesis, serving as an oncogenic factor or a tumor suppressor protein. Reducing macroH2A expression directly activates cyclin-dependent kinase 8 (CDK8), which promotes the development of malignant melanoma [134]. This suggests that the mechanism of macroH2A involvement in tumors is closely tied to its ability to regulate gene expression directly. MacroH2A isoforms have different roles in tumor development. MacroH2A2 suppresses the progression of carcinoma, including anal carcinoma, glioblastoma, and solitary dormant disseminated cancer [135,136,137]. Tumor self-renewal is a key feature of tumor proliferation. ATAC-seq and macroH2A2 ChIP-seq results showed that macroH2A2 alters chromatin accessibility, regulates enhancer progenitor function, and represses the expression of tumor growth genes, thus antagonizing tumor self-renewal and inhibiting glioblastoma growth [136]. Consistent with this result, overexpression of macroH2A2 inhibited tumor cell growth as well as metastasis, and transcriptomic results suggested that this might be related to its inhibition of the cell cycle and pro-tumor-related signaling pathways in solitary dormant disseminated cancer [137]. MacroH2A1.1 and macroH2A1.2 are often studied together in the same tumor types and they have different effects on various types of tumors. In most cases, macroH2A1.1 acts as a tumor suppressor, while macroH2A1.2 demonstrates a pro-tumorigenic role [138]. A downregulation of macroH2A1.1 and an elevated expression of macroH2A1.2 are detected in colon cancer. In vitro, macroH2A1.1 reduction increases tumor cell growth and proliferation [139]. A study reveals that macroH2A1.1, through its targeting of the PAR chain, acts as a suppressor of EMT in breast cells. Conversely, macroH2A1.2 showed no effect on EMT, and this partially elucidates the observed disparities in tumors [140]. However, the tumor promotion and inhibition functions of macroH2A change in different tumor contexts. MacroH2A1.2 reduces bone metastasis by attenuating the activity of lymphotoxin beta (LTβ) in prostate cancer and inhibiting secretion of lysyl oxidase (LOX) in breast cancer, thus exerting an anti-osteoclastogenic effect [89,141]. Interestingly, it has recently been shown that macroH2A1 participates in myelodysplastic syndromes (MDSs) by promoting NF-κβ-mediated inflammatory responses by regulating gene transcription [142]. These studies suggest that different macroH2A molecular mechanisms in different contexts lead to different results. However, what mediates this difference deserves further exploration. Notably, the ratio of macroH2A1.1/macroH2A1.2 assumes significance in cancer prognosis and can be modulated by pre-mRNA splicing regulators [138,143]. Splicing factors QKI enhances macroH2A1.1 expression, while RNA helicases DDX5/DDX17 augment macroH2A1.2 expression [138,143]. In prostate cancer, the expression of macroH2A1.1 and the pre-mRNA splicing regulator QKI is decreased [144], which indirectly proves their regulatory relationship. Considering the pivotal role of pre-mRNA splicing regulators, it is hypothesized that modulation of pre-mRNA splicing regulators could potentially impact the expression of various isoforms of macroH2A, consequently influencing tumorigenesis. This suggests that pre-mRNA splicing regulators present promising targets for the manipulation of macroH2A isoform expression, with significant implications for cancer therapy. The accurate targeting of specific macroH2A isoforms is crucial for effective disease management. However, the specific functions of different macroH2A isoforms in the pathogenesis of cancer remain inadequately elucidated. Therefore, there is a pressing need for further exploration of the involvement of macroH2A in tumorigenesis, as it has the potential to provide a more comprehensive theoretical foundation for the prevention and treatment of tumors.

**Table 1:** The role of histone H2A variants in cancer.

Histone Variants	Types of Cancer	Potential Mechanisms
H2A.Z	Lung cancer	Increases the sensitivity of lung cancer cells to radiotherapy [117].
	Breast cancer	Activates the expression of cyclin A1, thus promoting breast cancer development [37].
	Malignant melanoma	H2A.Z2 enhances tumor sensitivity to drugs by gene transcriptional regulation for E2F1 in malignant melanoma [119].
	Uterine leiomyoma	Deficient H2A.Z disposition in uterine leiomyoma cells with SRCAP complex mutations suggests a cancer inhibition role [118].
	Hepatocellular carcinoma	H2A.Z1 promotes tumorigenesis, mainly through cell cycle signaling and the DNA damage pathway [113], and H2A.Z acetylation promotes downstream pro-oncogene transcription in liver cancer [110].
	Bladder cancer	Promotes pro-oncogene expression in bladder cancer [120].
H2A.X	Gastrointestinal cancer	Inhibits EMT and promotes autophagy in colon cancer [123].
	Breast cancer	Increases Twist1 and Slug transcription factors, regulates EMT, and facilitates tumor cell migration and invasion after H2A.X deletion in breast cancer [125].
	Prostate cancer	Inhibits the proliferation of prostate cancer cells [111].
	Lung cancer	H2A.X deletion causes DNA breaks and cell cycle arrest in lung cancer [130].
	Head and neck carcinoma	A marker of DNA damage repair in head and neck carcinoma [131].
	Leukemia	Patients diagnosed with cancer usually occur in the absence of H2A.X in leukemia [133].
macroH2A	Malignant melanoma	Activates CDK8 to promote the development of malignant melanoma [134].
	Anal carcinoma	MacroH2A2 promotes the progression of anal carcinoma [135].
	Glioblastoma	MacroH2A2 antagonizes tumor self-renewal and inhibits glioblastoma growth [136].
	Solitary dormant disseminated cancer	nhibit cell cycle and pro-tumor-related signaling pathways in solitary dormant disseminated cancer [137].
	Breast cancer	IMacroH2A1.1 acts as a suppressor of EMT in breast cells [140].
	Colon cancer	Knockdown of macroH2A1.1 promotes tumor cell growth and proliferation in colon cancer [139].
	Hematologic malignancies	Lacking macroH2A1.1 induces the development of hematologic malignancies [145].
	Prostate cancers	MacroH2A1.2 is a tumor suppressor and inhibits osteoclast formation in prostate cancers [141].
H2A.B	Hodgkin’s lymphoma	Interacts with RNA polymerase II, increases ribosome biosynthesis in tumor cells, and promotes tumor development [112].

The conclusions on the effect of H2A histone variants, including H2A.Z, H2A.X, macroH2A, and H2A.B, in cancers.

### 3.2. Embryonic Development Abnormalities

**Figure 3:**
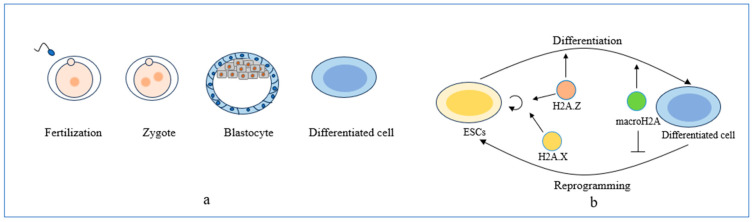
The role of histone H2A variants in embryonic development abnormalities. (**a**) The process of embryonic development. Embryonic development involves sequential stages: fertilization to form a zygote, development into a blastocyte, and differentiation into specialized cells (**b**) The role of H2A.Z, H2A.X, and macroH2A in embryonic development. H2A.Z promotes self-renewal as well as differentiation of ESCs. H2A.X promotes self-renewal of ESCs. MacroH2A promotes the differentiation of ESCs and inhibits the reprogramming of differentiated cells.

Histone H2A variants participate in embryonic development abnormalities (Figure 3b). It is imperative to recognize that abnormal embryonic development may result in congenital birth defects. Notably, data from 2019 illustrate that congenital birth defects stood as the fourth principal cause of mortality among children below the age of five, constituting nearly 10 percent of pediatric fatalities [146]. The process of a zygote developing into a metazoan is complicated since metazoans contain different cell types with the same gene sequence (Figure 3a). Epigenetic inheritance is crucial in the transcriptional regulation of genes during mammalian embryonic development [147]. As a key component of epigenetic inheritance, histone H2A variants are implicated in forming zygotes, cell differentiation, and embryo implantation [95,147]. Histone H2A variants exhibit distinct functions during embryonic development. Abnormal embryonic development often arises due to the dysregulated expression of histone variants [148,149,150]. Understanding the roles of histone H2A variants in embryonic development is crucial for identifying potential pathogenic mechanisms for developmental diseases.

H2A.Z participates in embryonic development abnormalities by influencing gene transcription activation related to pluripotency, differentiation, and heterochromatin formation [95]. H2A.Z knockout mouse embryos fail at implantation, leading to death [148], which indicates the important role of H2A.Z in early embryonic development, differentiation, and proliferation. H2A.Z promotes the self-renewal and differentiation of embryonic stem cells (ESCs). H2A.Z has been identified as a regulator of differentiation in various lineages, such as the endoderm [96], mesoderm [114], neural cells [97], muscle cells [99,151], melanocytes [152], and intestinal cells [153]. H2A.Z is enriched in the promoter and enhancer regions of chromatin in mouse ESCs, which creates conditions for H2A.Z to regulate the expression of genes related to mouse ESCs. H2A.Z deposition increases chromatin accessibility, whereas knockdown of H2A.Z inhibits the expression of OCT4-related target genes and the expression of differentiation-related genes, thus affecting the self-renewal of genes and the differentiation of cells [154]. This suggests that H2A.Z plays a major role in promoting gene expression during ESCs growth and differentiation. The process of transcriptional quiescence to the active genes is identified as Zygotic Genome Activation (ZGA) in early embryonic development, and is essential for embryonic development. When the ZGA process is abnormal, the embryo fails to develop into a normal embryo [155]. H2A.Z exhibits low expression levels during ZGA in mice [156]. A recent study shows that decreasing H2A.Z enrichment at the TSS is correlated with the downregulation of housekeeping genes at ZGA in *Drosophila*, leading to chromatin structure alterations and abnormalities in embryonic development [157]. Current studies have not addressed the role of H2A.Z in the mammalian ZGA phase. H2A.Z is conserved in most species [158,159]. Therefore, we speculate that H2A.Z likely plays the same role in mammals. However, further exploration is necessary to understand this mechanism. It is worth noting that H2A.Z is an essential regulator of the EMT process [114], a crucial event of embryo implantation and prometaphase formation, and mediates the generation of the mesoderm [160]. Although both H2A.Z and EMT play essential roles in embryonic development, no study has yet demonstrated that H2A.Z directly mediates embryonic development by mediating EMT. In conclusion, H2A.Z plays a predominantly promotional role in embryonic development, which raises the question of whether H2A.Z also plays a repressive role. In addition, further research is required to determine whether H2A.Z influences embryonic development via EMT.

The involvement of H2A.X in embryonic development centers on ESCs [161]. Unlike H2A.Z, mice remained viable after the knockout of H2A.X. However, they exhibit stunted growth, reduced fertility, and decreased lymphocytes, resulting in immune deficiencies [149]. Simultaneous depletion of H2A.X and ATM results in embryonic lethality in mice, revealing the crucial involvement of H2A.X during embryogenesis [162]. The deposition of H2A.X into chromatin contributes to maintaining self-renewal and proliferation in ESCs. Despite low levels of DNA damage repair, mouse ESCs exhibit remarkably elevated basal levels of γ-H2A.X [163]. This observation indicates other mechanisms for γ-H2A.X to participate in embryonic development beyond its canonical role in DNA damage repair. In particular, H2A.X is deposited in the promoter region of rDNA in mouse ESCs, recruiting nucleolus remodeling complexes and inhibiting rDNA transcription to restrict ESC proliferation [164]. This indicates that the involvement of H2A.X in the growth of ESCs is partially dependent on gene transcription. In addition to its role in ESCs, H2A.X also significantly influences somatic stem cells. A recent study has demonstrated that γ-H2A.X regulates the self-renewal and differentiation of human pluripotent stem cells and leukemic progenitors [82]. Deletion of γ-H2A.X promotes differentiation of pluripotent stem cells to hematopoietic stem cells and inhibits their differentiation to neuronal development [82]. Furthermore, H2A.X regulates extraembryonic genes. Research indicates that the deposition of H2A.X is crucial for maintaining the level of H3K9me3 at enhancer sites of extraembryonic genes [165]. The available evidence strongly suggests that γ-H2A.X serves a critical function in regulating gene transcription. However, the precise molecular mechanism by which H2A.X modulates gene transcription remain poorly defined and warrant further exploration. In light of the multifaceted roles of H2A.X in embryonic developmental disorders, a comprehensive understanding of these mechanisms is essential. Thus, additional studies are required to elucidate the underlying biological pathways and their interactions.

Studies on the role of macroH2A in embryonic development are limited, and most focus on its role in later stages of cell differentiation. In mice deficient in macroH2A, growth and development are restricted [150,166]. MacroH2A exhibits low expression levels during mouse embryogenesis while establishing pluripotency in early ESCs [167], which further suggests that macroH2A primarily exerts function in late growth and development in mammals. CHIP results showed that macroH2A is up-regulated and recruited to cell differentiation regulatory genes marked with H3K27me3, promoting gene expression suppression during cell differentiation [168]. It has been demonstrated that macroH2A impedes the reprogramming of somatic cells to pluripotency in humans [168]. All of the above confirms the role of macroH2A in late development. Interestingly, it has been shown that macroH2A also plays a role in early embryonic development. In *zebrafish* embryos, macroH2A1 and macroH2A2 are distinctly localized in the genome. RNA-seq data shows macroH2A1 is critical in downregulating gene expression in specific cells and embryonic stages, and its impact is linked to nuclear quiescence. In contrast, macroH2A2 is associated with upregulating differentially expressed genes during embryonic development [169]. The findings of these studies suggest a different function between different isoforms of macroH2A in the early stage of embryonic development in *zebrafish*. Before embryo implantation, macroH2A1 accumulation marks inactivation with the X chromosome in mice [170]. This observation implies that macroH2A could potentially exert influence during the early stages of mammalian embryonic development. Furthermore, it has been proven that macroH2A is recruited to regulatory regions of pluripotency and developmental genes that regulate cell differentiation during the latter stage of mouse embryonic cell differentiation. Knockdown of macroH2A1 or macroH2A2 significantly enhances the efficiency of induced pluripotency in mice [167]. These findings diverge from the observed function of macroH2A2 in *zebrafish* outlined earlier. This discrepancy may be attributed to interspecies variation as well as disparate developmental stages. The existing understanding of the mechanism underlying the involvement of macroH2A in embryonic development predominantly stems from research conducted on *zebrafish*. In contrast, investigations into the role of macroH2A in mammals have primarily focused on somatic stem cells. Consequently, additional studies are required to elucidate the potential contribution of macroH2A in mammalian embryonic development.

Little research exists on H2A.B in embryonic development. A comprehensive genome-wide analysis of H2A.B in mouse ESCs has revealed its association with methylated DNA within gene body regions [171], exerting a positive regulatory influence on transcription elongation. Knocking out all three H2A.B genes in male mice results in an altered chromatin structure in mouse germ cells and reduced embryo survival [172]. This reflects the potential importance of H2A.B in embryonic development. Subsequent investigations are necessary to elucidate the potential contributions of H2A.B to embryonic development.

### 3.3. Other Diseases

In addition to cancer and embryonic developmental disorders, histone H2A variants are also involved in other physiological processes such as muscle regeneration disorders [99], cellular senescence [100,101], neurological diseases [97,98], metabolic disorders [102,103,104] and cardiovascular diseases [105,106,107] by affecting gene expression. The histone variants H2A.Z, H2A.X, and macroH2A have been extensively studied in the context of neurological and psychiatric disorders. Neurological and psychiatric disorders are highly prevalent diseases affecting populations worldwide. In 2021, an estimated 340 million individuals experienced neurological health deficits and 111.1 million succumbed to neurological disorders [173]. Aberrations in neurogenesis have been identified as one of the underlying causes of a range of neurological diseases. H2A.Z is a negative regulator of memory preservation in the hippocampal region, and the administration of lentiviral knockdown of H2A.Z increases the memory of mice compared with control. Transcriptomic analysis showed that H2A.Z alters the expression of multiple memory-related genes [174]. This study endeavors to investigate the potential role of H2A.Z in the process of memory consolidation. Nevertheless, the precise mechanism by which H2A.Z modulates alterations in the hippocampal structure within the central nervous system is yet to be elucidated. Interestingly, T et al. showed that H2A.Z regulates embryonic neurogenesis and memory by promoting Nkx2-4 transcription through interaction with Setd2 [97], which links embryonic neurodevelopment to the neurological deficits in mice. In particular, the histone chaperone Anp32e of H2A.Z affects learning and memory in mice and influences the morphology of dendritic cells in cultured hippocampal neurons by removing H2A.Z from chromatin [175]. H2A.Z acetylation is elevated in schizophrenic patients compared to a control group. As an H2A.Z acetylation reader, BRD4 mediates the onset and progression of schizophrenia, suggesting that targeting BRD4 or H2A.Z acetylation promises to be a potential treatment for schizophrenia [176]. The above results indicate that H2A.Z is important in regulating neurological and psychiatric disorders. Histone chaperones and PTMs of H2A.Z mediate the development of diseases by affecting H2A.Z. H2A.X is also studied in neurogenesis. H2A.X-deficient mice exhibit reduced neuronal cellularity. Mitochondrial damage exacerbates neuronal cell destruction. H2A.X safeguards neuronal cells by regulating mitochondrial homeostasis [177]. Consistent with this, Weyemi et al. found that the knockdown of H2A.X in mice impaired learning behavior and homeostasis, while treatment with ROS inhibitors improved symptoms [178]. These mechanisms are linked to H2A.X as a regulator for DNA damage repair. H2A.X also acts as a promoter for the differentiation of human stem cells. Given this information, it is pertinent to investigate whether H2A.X participates in neurogenesis through this particular mechanism. MacroH2A1 is integrated into the chromatin structure of regulatory regions associated with pluripotency genes in adult neural stem cells [167]. The absence of macroH2A1.2 in mice leads to the manifestation of Autism Spectrum Disorder (ASD). The underlying mechanism is partially attributed to the reduction in NKX2.2 expression following the knockdown of macroH2A1.2, which subsequently hampers the proliferation of neural progenitors and promotes neuronal differentiation [167]. In addition, studies have shown that PARP1 is involved in neurodegenerative diseases [179]. However, there are no studies of macroH2A mediating neurological disorders through PARP1. Further investigation is warranted to elucidate the potential involvement of macroH2A in neurological disorders through its impact on PARP1.

MacroH2A is implicated in metabolic diseases by mediating adipogenesis. There are contradictory findings concerning the role of macroH2A isoforms in the regulation of adipogenesis. It has been demonstrated that the overexpression of macroH2A1.1, but not macroH2A1.2, reduces lipid accumulation in hepatocytes [102]. In contrast, one study shows that increased expression of macroH2A1.1 activates the Wnt/β-catenin gene transcription by interacting with the histone H3K27 methyltransferase EZH2, promoting adipogenesis and contributing to obesity occurrence in 3T3-L1 cells [103]. Another study discovered that macroH2A1.2 inhibits adipogenesis. In the macroH2A1.2 transgenic mouse model, adipogenesis was inhibited and liver and pancreatic damage caused by obesity was reduced. Transcriptome analysis shows a decrease in adipogenesis promoting genes and an increase in anti-inflammatory genes [104]. The variation in findings could potentially be attributed to the unique cellular and tissue compositions involved. The mechanism of macroH2A isoforms in metabolic disease needs more studies to be validated. H2A.X is involved in metabolism by mediating mitochondrial function [177] and oxidative stress processes [178] in neurological diseases. However, the potential involvement of H2A.X in the etiology of related diseases through its impact on metabolic processes remains an unresolved question. In addition, aberrant expression of H2A.B was observed in diabetic oocytes [180], which indicates that H2A.B may be essential in metabolic diseases. Of these histone H2A variants, only the role of H2A.Z in metabolic diseases has not yet been mentioned. Considering its significant impact on gene expression, it is plausible that H2A.Z is also involved in metabolic diseases.

Emerging evidence strongly suggests a significant correlation between histone H2A variants and the incidence of cardiovascular diseases. H2A.Z is elevated in cardiac hypertrophy [105]. Znhit1 is a major subunit of the SRCAP complex. A lack of Znhit1 is implicated with arrhythmias and heart failure occurrence. It has been demonstrated that Znhit1 plays a crucial role in maintaining the heart’s normal function by regulating the deposition of H2A.Z in the promoter region [105]. Moreover, Anp32e regulates the phosphorylation of H2A.Z through the activity of PP2A, thereby influencing gene transcription involved in the growth induction in cardiac cells. This finding highlights the potential role of Anp32e and PP2A in regulating cardiac growth and suggests a potential therapeutic target for cardiovascular diseases [36]. In cardiomyocytes, exogenous H2O2-induced oxidative stress increases the phosphorylation of H2A.X [106]. Phosphorylated H2A.X in circulating peripheral blood mononuclear cells in people with high metabolic syndrome risk is elevated compared with healthy individuals, which suggests H2A.X may serve as a predictor of cardiometabolic risk [107]. However, it requires further studies to determine whether H2A.X is a potential predictor of cardiovascular diseases, and whether it is used to effectively predict other diseases is worth exploring.

## 4. Conclusions

The replacement of canonical histones by histone H2A variants is a pivotal process in gene regulation and DNA damage repair. This phenomenon forms the basis for potential involvement in various pathophysiological processes associated with several diseases. The roles of histone H2A variants in gene transcription and DNA damage repair have been extensively described, as illustrated in Figure 1 and Figure 2. H2A.Z plays a dual role in both repressing and activating gene transcription. The mechanisms of its function are complex and mainly related to the stability of the nucleosome and the degree of H2A.Z accumulation in the TSS region. H2A.Z is also involved in DNA damage repair. The function of H2A.B is primarily to facilitate gene transcription and regulate RNA splicing. MacroH2A represses gene expression, which is related to chromatin condensation. In addition, macroH2A is involved in DNA damage repair, and different macroH2A variants perform distinct functions. H2A.X is a crucial factor that promotes DNA damage repair. γ-H2A.X amplifies DNA damage signaling and thus facilitates the aggregation of damage repair factors.

Histone H2A variants participate in the progression of various diseases, including cancer, embryonic developmental diseases, neurological diseases, metabolic diseases, and cardiovascular diseases (Table 2). H2A.Z and macroH2A promote or suppress cancer proliferation. H2A.X mainly suppresses tumorigenesis, and H2A.B has also been confirmed to be associated with cancer. The histone H2A variants are significant contributors to embryonic development. Their aberrant expression has been linked to embryonic developmental diseases, as well as a range of neurological and psychiatric diseases. Notably, the H2A.Z and H2A.X variants have been implicated in cardiovascular diseases, and the macroH2A variant is also associated with metabolic diseases. These findings highlight the potential importance of detecting the expression of histone H2A variants in the context of disease pathology.

Notwithstanding the considerable progress made in the study of histone variants, several inquiries remain unresolved, warranting further investigation. How does H2A.Z fulfill dual functions in gene activation and silencing? H2A.X occupies a pivotal position in the mechanisms of DNA damage repair, whereas it also activates the fibrotic pathway and is crucial for embryonic growth and development. Interestingly, these roles might operate beyond H2A.X’s primary responsibilities related to DNA damage repair. Further investigation is required to elucidate the mechanisms underlying the role of H2A.X in gene transcription. Additionally, the inconsistent functions of various subtypes of macroH2A in the context of cancer and metabolic diseases necessitate further exploration. The role of H2A.B is primarily to facilitate gene transcription, while H2A.B is widely present in DNA damage sites and replication sites. Furthermore, H2A.B is capable of triggering the NF-κβ pathway, playing a role in DNA damage repair. This suggests a possible involvement of H2A.B in DNA damage repair through modulation of gene transcription activities. However, the extent to which H2A.B may participate in the modification of DNA damage, independent of its transcriptional regulatory functions, merits further scientific investigation. In addition, the mechanisms by which histone chaperones regulate nucleosome assembly and turnover and their roles in mediating gene expression and disease development warrant further study. The participation of H2A.B in the EMT process remains uncertain despite the established roles of H2A.Z, H2A.X, and macroH2A in this process. Moreover, how histone H2A variants coordinate with each other to be involved in the pathophysiological processes of disease deserves further investigation. In the future, extensive investigations into the functional relevance of histone H2A variants have the potential to significantly augment their clinical utility in the domains of disease diagnosis, prediction, and treatment. In conclusion, it is recommended that future research delve deeper into the involvement of H2A histone variants in gene transcription and DNA damage repair. Furthermore, it is imperative to explore the roles and mechanisms of H2A histone variants in diseases beyond cancer.

**Table 2:** The conclusions on H2A histone variants.

Histone H2A Variants	Distribution	Isoforms	Role in Gene Transcription	Role in DNA Damage Repair	Pathophysiological Processes Involved	The Roles in Disease
H2A.Z	Global	H2A.Z1 H2A.Z2 H2A.Z2.2	Gene transcription activation or inhibition [32,33]. The mechanism is associated with the stability of nucleosomes [32], enhancer activity [25], RNA polymerase II pausing [29,31], and PTMs of H2A.Z nucleosomes [93,181,182].	Promotes DNA repair [60,61,63]. Removing from nucleosomes recruiting repair factors to the break region and promoting DNA damage repair [61,63].	Tumorigenesis [37,110,117] Embryonic development disorders [25,95,148] Neurological diseases [97,98] Heart diseases [105]	Promotes tumorigenesis [37,110,117]. Inhibits tumorigenesis in uterine leiomyoma cells with SRCAP complex mutations [118]. H2A.Z knockout mouse embryos die [148]. Regulates neurogenesis [97,98], affects learning and memory [175], and mediates the progression of schizophrenia [176]. Cardiac hypertrophy [105] and regulation of cardiac growth [36].
H2A.X	Global		Transcription related [81]. The phosphorylation H2A.X axis mediates TGFB1-associated gene transcription activation, aggravating pulmonary fibrosis [81].	Accelerates DNA damage repair [6,71] γ-H2A.X amplifies the signal and functions as a platform for assembling the DNA damage repair machinery [6,71].	Tumorigenesis [93,125,126] Embryonic development disorders [82] Neurological diseases [177] Heart diseases [106,107] Metabolic diseases [177]	Inhibits tumorigenesis [93,125,126]. H2A.X knockout mice exhibit reduced fertility and a decreased number of lymphocytes, resulting in immune deficiencies [149]. Depletion of H2A.X causes neurological disorders [177,183]. Aberrant expression in heart diseases [106,107]. Mediating mitochondrial function [177] and oxidative stress processes [178].
macroH2A	Global	macroH2A1.1 macroH2A1.2 macroH2A2	Mostly represses gene transcription [50]. The mechanism is associated with RNA polymerase II initiation [53], chromatin remodeling [54], histone acetylation [56], transcription factors activity [184], and chromatin condensation [50].	Promotes DNA damage repair. Required for transcriptional repression near breaks following DNA damage [50,83,84,90] MacroH2A1.2 recruits DNA damage repair mediators [84]. MacroH2A1.1 impedes the activity of PARP1 [83,85].	Tumorigenesis [94,135,138] Embryonic development disorder [150,166] Neurological diseases [179] Metabolic diseases [102,104]	Promotes tumorigenesis or inhibits tumorigenesis [94,135]. MacroH2A-deficient mice’s growth and development are restricted [150]. The absence of macroH2A1.2 in mice leads to the manifestation of ASD [179]. Promotes adipogenesis [104]. Reduces lipid accumulation in hepatocytes [102].
H2A.B	Mainly in the testes and brain		Promotes gene transcription [19,20]. H2A.B nucleosomes are poorly stable, resulting in easy dissociation from chromatin, the formation of open chromatin structures, and gene transcription activation [19,20].	Causes DNA damage [48] H2A.B overexpression in Hela cells induces DNA damage and subsequent apoptosis by activating the NF-κβ pathway [48].	Tumorigenesis [112] Embryonic development disorders [42] Metabolic diseases [180]	Promotes tumor development [112]. The viability of embryos from H2A.B knockout male mice mated with female mice is reduced [172]. Aberrant expression in diabetic oocytes [180].

The conclusions on H2A histone variants, which includes H2A histone variants’ distribution, isoforms, roles in gene transcription and DNA damage repair, the pathophysiological processes involved, and their roles in diseases.

## Data Availability

All data are incorporated into the article.
